# Beat-to-Beat Variability of Ventricular Action Potential Duration Oscillates at Low Frequency During Sympathetic Provocation in Humans

**DOI:** 10.3389/fphys.2018.00147

**Published:** 2018-04-04

**Authors:** Bradley Porter, Stefan van Duijvenboden, Martin J. Bishop, Michele Orini, Simon Claridge, Justin Gould, Benjamin J. Sieniewicz, Baldeep Sidhu, Reza Razavi, Christopher A. Rinaldi, Jaswinder S. Gill, Peter Taggart

**Affiliations:** ^1^Department of Imaging Sciences and Biomedical Engineering, Kings College London, London, United Kingdom; ^2^Guy's and St Thomas' Hospital, London, United Kingdom; ^3^Department of Cardiovascular Sciences, University College London, London, United Kingdom

**Keywords:** arrhythmia, sympathetic nervous system, oscillations, action potential duration variability, activation recovery interval

## Abstract

**Background:** The temporal pattern of ventricular repolarization is of critical importance in arrhythmogenesis. Enhanced beat-to-beat variability (BBV) of ventricular action potential duration (APD) is pro-arrhythmic and is increased during sympathetic provocation. Since sympathetic nerve activity characteristically exhibits burst patterning in the low frequency range, we hypothesized that physiologically enhanced sympathetic activity may not only increase BBV of left ventricular APD but also impose a low frequency oscillation which further increases repolarization instability in humans.

**Methods and Results:** Heart failure patients with cardiac resynchronization therapy defibrillator devices (*n* = 11) had activation recovery intervals (ARI, surrogate for APD) recorded from left ventricular epicardial electrodes alongside simultaneous non-invasive blood pressure and respiratory recordings. Fixed cycle length was achieved by right ventricular pacing. Recordings took place during resting conditions and following an autonomic stimulus (Valsalva). The variability of ARI and the normalized variability of ARI showed significant increases post Valsalva when compared to control (*p* = 0.019 and *p* = 0.032, respectively). The oscillatory behavior was quantified by spectral analysis. Significant increases in low frequency (LF) power (*p* = 0.002) and normalized LF power (*p* = 0.019) of ARI were seen following Valsalva. The Valsalva did not induce changes in conduction variability nor the LF oscillatory behavior of conduction. However, increases in the LF power of ARI were accompanied by increases in the LF power of systolic blood pressure (SBP) and the rate of systolic pressure increase (dP/dt_max_). Positive correlations were found between LF-SBP and LF-dP/dt_max_ (*r*_s_ = 0.933, *p* < 0.001), LF-ARI and LF-SBP (*r*_s_ = 0.681, *p* = 0.001) and between LF-ARI and LF-dP/dt_max_ (*r*_s_ = 0.623, *p* = 0.004). There was a strong positive correlation between the variability of ARI and LF power of ARI (*r*_s_ = 0.679, *p* < 0.001).

**Conclusions:** In heart failure patients, physiological sympathetic provocation induced low frequency oscillation (~0.1 Hz) of left ventricular APD with a strong positive correlation between the LF power of APD and the BBV of APD. These findings may be of importance in mechanisms underlying stability/instability of repolarization and arrhythmogenesis in humans.

## Introduction

Factors which modulate or destabilize ventricular repolarization are of fundamental importance in arrhythmogenesis. Spontaneous beat-to-beat fluctuation in repolarization is an inherent property of ventricular myocardium (Baumert et al., 2016). Beat-to-beat variability (BBV) of repolarization although influenced by cycle length (Boyett and Jewell, 1978) is largely due to variation in action potential duration (APD) (Zaniboni et al., 2000) and usually measured in humans as QT interval variability (Tereshchenko et al., 2009; Hinterseer et al., 2010). Enhanced BBV has been shown to be associated with arrhythmia in a range of animal models (Thomsen et al., 2004; Gallacher et al., 2007; Abi-Gerges et al., 2010; Jacobson et al., 2011) and humans (Atiga et al., 1998; Haigney et al., 2004; Tereshchenko et al., 2009; Hinterseer et al., 2010; Sredniawa et al., 2012) and has been proposed as an adjunct to clinical assessment for implantable cardioverter defibrillator (ICD) implantation (Baumert et al., 2016).

Provocations which increase sympathetic activity have been shown to enhance BBV of repolarization (Desai et al., 2004; Piccirillo et al., 2006; Johnson et al., 2013; Porter et al., 2017). In neural signals information is coded simultaneously using two different modalities, i.e., amplitude strength or “tonic activity” and the discharge pattern, i.e., oscillation or phasic patterning (Gerstner et al., 1997; Coote, 2001; Montano et al., 2009). Recently attention has been drawn to both the physiological and clinical importance of the phasic nature of sympathetic nerve activity in arrhythmogenesis (Rizas et al., 2014, 2016, 2017). Sympathetic nerve activity is organized in a series of low frequency bursts and it has been shown that low frequency rhythmic modulations of repolarization can be identified from the T-wave vector in the ECG which are associated with sympathetic activity (Rizas et al., 2014, 2016). When pronounced, these oscillations have been shown to be one of the strongest predictors of ventricular arrhythmia and sudden cardiac death in post myocardial infarction patients (Rizas et al., 2017). These oscillations are considered to reflect oscillations of ventricular APD. We have identified oscillations of ventricular APD in the low frequency range in humans under conditions of enhanced sympathetic activity (Hanson et al., 2014); under conditions of calcium load and reduced potassium currents common in pathological hearts, such oscillations may be arrhythmogenic by the generation of after depolarizations (Pueyo et al., 2016). In addition, increased BBV may further contribute to destabilization of repolarization that may facilitate re-entry arrhythmias.

In the present study, we have examined the hypothesis that in heart failure patients an acute sympathetic challenge may not only induce low frequency oscillatory behavior of ventricular APD but that by inducing low frequency oscillations there is a resultant increase in the magnitude of APD variability. We hypothesized that these changes would be accompanied by similar changes in the low frequency behavior of arterial blood pressure (Mayer waves; Julien, 2006).

## Methods

### Ethical approval

The study was approved by the West London Ethics Committee and conformed to the standards set by the Declaration of Helsinki (latest revision: 64th WMA General Assembly). Informed consent was obtained in writing from all subjects.

### Subjects

Studies were performed in 11 ambulatory heart failure patients (all male, age 58–76) who were recipients of a cardiac resynchronization therapy defibrillator (CRT-D) device (Quadra Assura MP™ CRT-D, St. Jude Medical). Patient characteristics are shown in Table 1. Exclusion criteria were inherited channelopathies, hypertrophic cardiomyopathy and use of Class I or III antiarrhythmics.

**Table 1:** Patient characteristics.

**Characteristics**	
Ischaemic cardiomyopathy, *n* (%)	6 (54.5)
Ejection fraction ±*SD*, %	35.3 ± 13.4
NYHA class 1, *n* (%)	2 (18.2)
NYHA class 2, *n* (%)	7 (63.6)
NYHA class 3, *n* (%)	2 (18.2)
Diabetes mellitus, *n* (%)	2 (18.2)
Atrial fibrillation, *n* (%)	2 (18.2)
Beta-blockade, *n* (%)	8 (72.7)
ACE inhibitor, *n* (%)	9 (81.8)
Aldosterone antagonist, *n* (%)	7 (63.6)

*NYHA, New York Heart Association*.

### Physiological recordings

**Figure 1:**
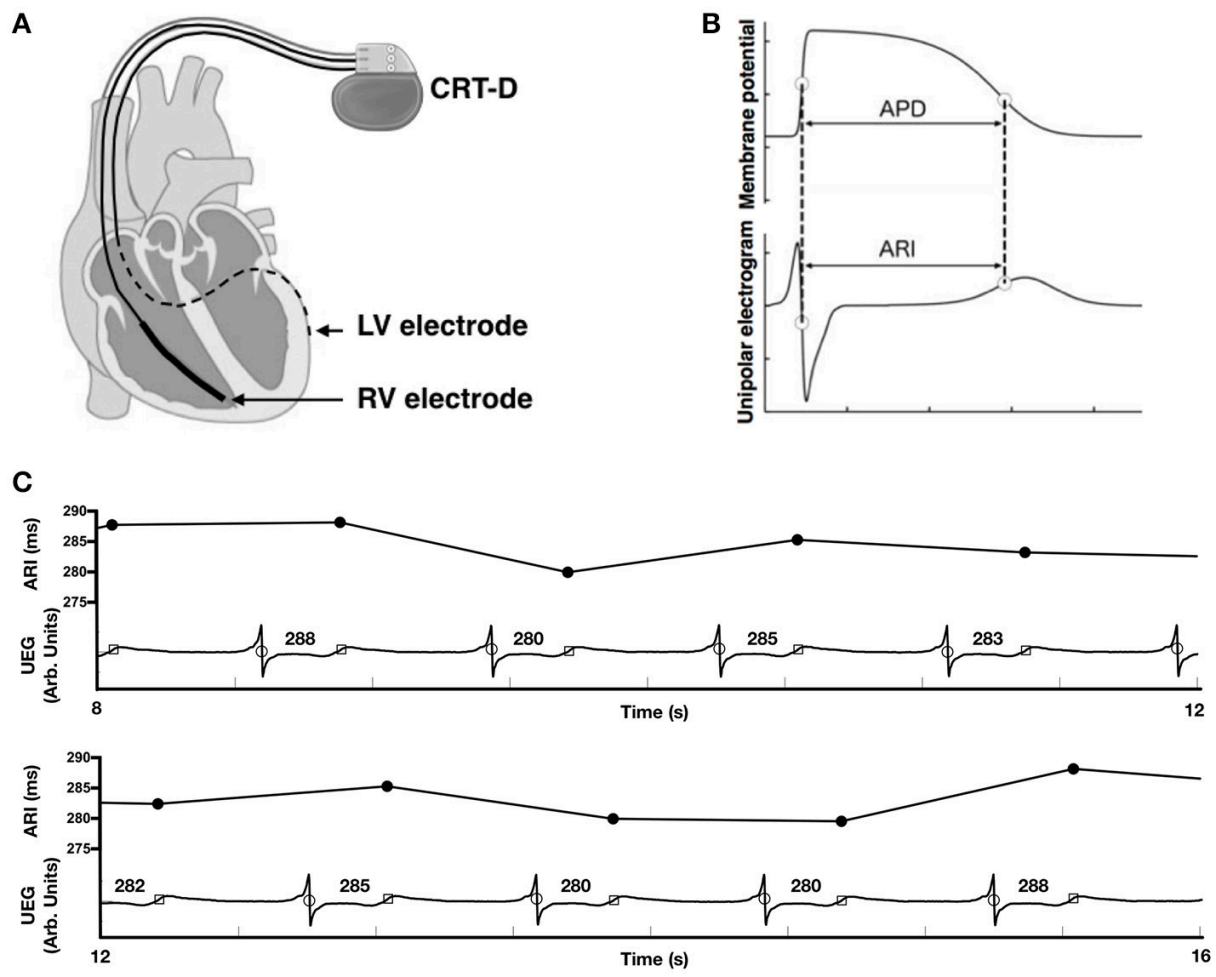
**(A)** Cardiac resynchronization therapy defibrillator (CRT-D) device programmed to pace from the right ventricular (RV) electrode and record a local unipolar electrogram (UEG) from the left ventricular (LV) electrode. **(B)** Relationship between the unipolar electrogram and the intracellular ventricular transmembrane potential showing correspondence between activation recovery interval (ARI) and action potential duration (APD). **(C)** Example UEG recorded from the left ventricular lead and the computed ARI values for the same electrogram trace.

The implanted CRT-D device was used to record unipolar electrograms (UEGs) from the left ventricular epicardial lead, sampled at 512 Hz (Figure 1) (Hanson et al., 2014; Chen et al., 2016). The devices used in this study allowed storage of five separate recordings of UEGs at 30 s duration. Synchronized simultaneous recordings of arterial blood pressure were made non-invasively using a finger cuff (Finometer pro, Finapres Medical Systems B.V., Amsterdam, The Netherlands) (Imholz et al., 1988). The signals were digitized by the MP150 System using AcqKnowledge software (Goleta, CA) and sampled at 1 kHz. Simultaneous breathing activity was recorded using a respiration transducer and respiration pneumogram amplifier (TSD201 and RSP100C, Biopac Systems Inc., Goleta, CA). The respiration signal was used to confirm the end of the Valsalva and the absence of low frequency respiration which could potentially generate low frequency oscillation in ARI (Hanson et al., 2012).

### Protocol

Following recruitment to the study, beta-adrenergic blocking agents (bisoprolol) were discontinued for 5 days prior to the start of the protocol to allow for a sufficient wash-out period (Leopold et al., 1986). Whilst seated upright the following stages took place:

1. Fixed cycle length was achieved through right ventricular pacing using their implanted CRT-D device. Right ventricular pacing was chosen over atrial pacing to account for a high prevalence of AF within our heart failure population and therefore standardize protocols between patients (Bueno-Orovio et al., 2014). The rate was chosen as the minimum rate required to maintain continuous capture at a fixed cycle length. A minimum adaptation period of 10 min took place prior to any recordings (Franz et al., 1988).
2. With the patient at rest, recordings of UEG, blood pressure and respiration took place. UEG data was then extracted from the device programmer to allow further recordings in step 3.
3. Subjects were asked to perform the Valsalva maneuver (forced expiration against a fixed resistance; Doytchinova et al., 2016) for 10 s. The maneuver was conducted ~35 s into the second batch of UEG recordings. Blood pressure and respiration recordings were made continuously before, during and after the procedure.

The Valsalva maneuver is an established method of increasing sympathetic activity whereby forced expiration impedes venous return resulting in a reduction in ventricular pressure and volume, and a baroreflex increase in sympathetic activity (Booth et al., 1962; Korner et al., 1976; Smith et al., 1987). Sympathetic activity has been shown to be greatly enhanced during the strain phase of the Valsalva maneuver in healthy control subjects in studies using microneurographical recordings of muscle sympathetic nerve discharges (Schrezenmaier et al., 2007). Indices of baroreflex sensitivity have been established to separately evaluate the vagal and adrenergic components (Vogel et al., 2005; Schrezenmaier et al., 2007).

### Analysis of data

#### Raw data

Raw digital UEG traces were analyzed off-line using custom built MATLAB software (MathWorks Inc., Natick, Mass) as described previously (Chen et al., 2013, 2016). Each 30 s output from the CRT-D device was truncated to form one long data sequence of ~150 s in length. Any possible overlap between successive 30 s sequences was found by searching for matching traces at the start/end of successive traces and was removed. Activation recovery intervals (ARIs) were measured from the time of minimum dV/dt of the electrogram QRS complex, representing local activation time (AT), to the time of maximum dV/dt of the subsequent T-wave, representing local repolarization time (RT) (Wyatt et al., 1981; Haws and Lux, 1990; Coronel et al., 2006; Potse et al., 2009; Hanson et al., 2012, 2014). Figure 1C shows an example of the identification of the AT and RT and resultant ARI for each complex. Blood pressure recordings were analyzed for systolic blood pressure (SBP) and the maximum rate of systolic pressure increase (dP/dt_max_) for each beat using a script written in MATLAB (Mathworks, Inc., Natick, MA, USA). Ectopic beats (0.6 ± 0.8% of beats across all recordings) were removed from analysis together with the successive beat.

#### Beat-to-beat variability analysis

We have previously demonstrated a transient temporal increase in the short-term variability of ARI immediately following the Valsalva using a 10-beat moving window (Porter et al., 2017). Here our analysis focusses on longer time periods of recording to allow for frequency domain analysis. As such the immediate 60 s following termination of the Valsalva was compared with the resting recordings taken prior to the onset of the Valsalva. The respiration recordings were used to determine the timing of the termination of the Valsalva.

Beat-to-beat variability of ARI was computed over the entire 60 s period as per established QT variability measures (Baumert et al., 2016). The standard deviation of ARI (SDARI) was computed as:

$$SDARI=\sqrt{\frac{1}{N}\sum {\left(ARI_{n}-ARI_{mean}\right)}^{2}}$$

the ARI variance normalized to the square mean ARI (nSDARI) was computed as:

$$nSDARI=\frac{SDARI^{2}}{AR{I_{mean}}^{2}}$$

the standard deviation of SBP (SD-SBP) and dP/dt_max_ (SD-dP/dt_max_), and the normalized variability values of SBP (nSD-SBP) and dP/dt_max_ (nSD-dP/dt_max_) were computed with the same formula as for ARI. The same formula was applied to AT-AT intervals to assess for any evidence of a change in conduction variability generated by the Valsalva.

#### Spectral analysis

**Figure 2:**
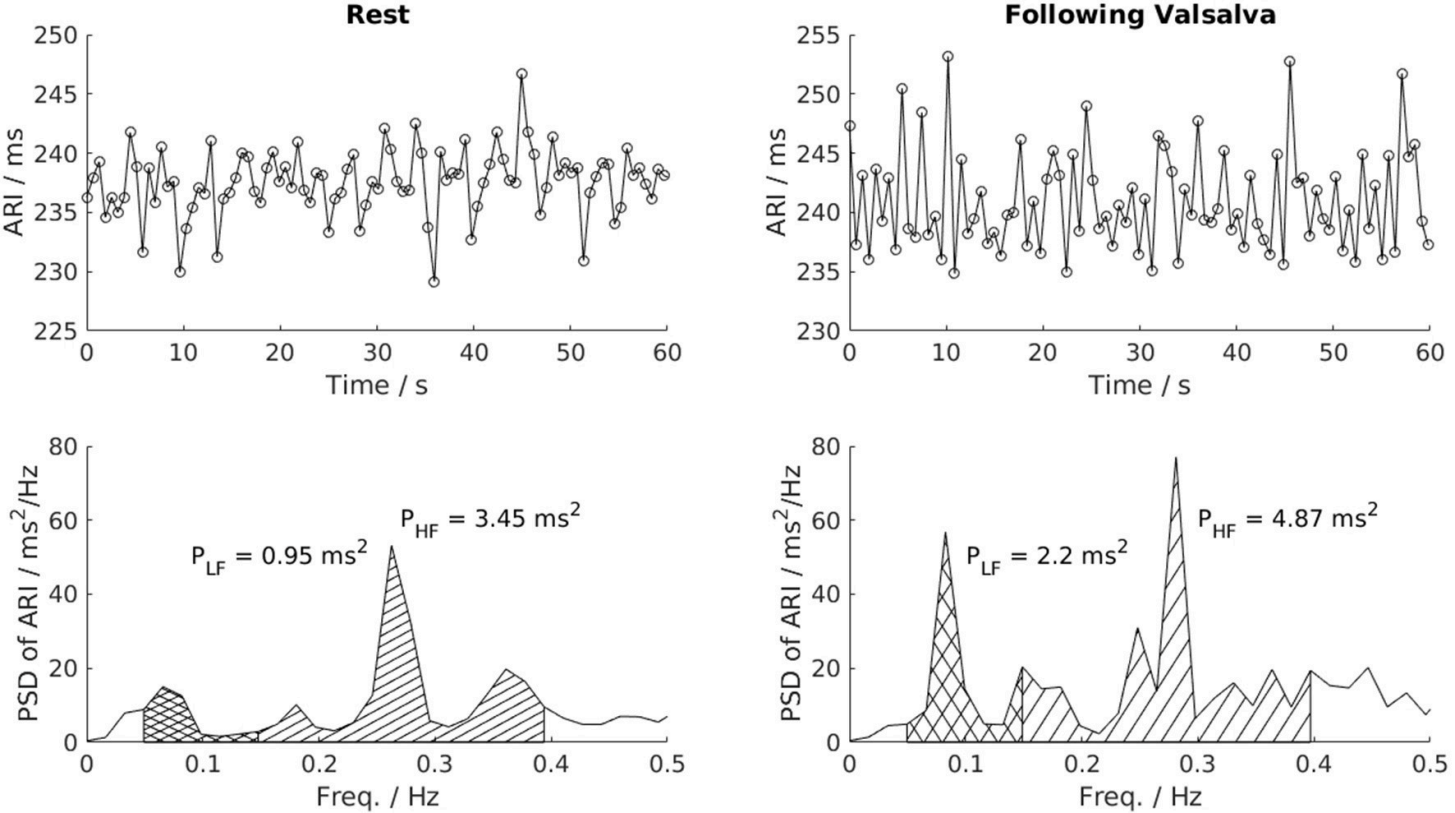
Power spectral analysis of low-frequency (LF, 0.04–0.15 Hz) and high-frequency (HF, 0.15–0.4 Hz) variability. This example shows a clear increase of LF variability of ARI in one patient following the Valsalva. PSD, Power spectrum density; P_LF_, low frequency power; P_HF_, high frequency power.

Spectral analysis was performed over the same 60 s recordings used to compute BBV measures. Before spectral analysis was performed, linear interpolation was applied to fill in missing AT-AT interval, ARI or blood pressure values (Malik, 1996). An auto-regressive model fitted to each AT-AT interval, ARI and blood pressure segment was computed using the Yule–Walker method. Following the recommendation of Kay (Kay, 1999), model orders were tested in the range L/3 to L/2, with L the number of beats. The optimal model order was chosen as that which minimized Akaike's Information Criterion (Akaike, 1974) and the residuals were required to pass whiteness test. The low and high frequency variability (LF and HF, respectively) in AT-AT interval, ARI, SBP and dP/dt_max_ were then calculated by integrating the band power across the bandwidth 0.04–0.15 Hz for LF and 0.15–0.4 Hz for HF. The normalized LF and HF variability (nLF and nHF, respectively) were also calculated by dividing the LF and HF variability by the total power in the range above 0.04 Hz (“normalization power”), as recommended by Malik (1996). Both markers represent the power of the LF and HF component relative to the total power of the spectrum. As sympathetic activity is organized in a series of low frequency bursts, the LF and normalized LF are used to expose changes in sympathetic activity. Figure 2 shows the construction of the LF and HF band in the spectrum and the corresponding changes in LF and HF power in the ARI series of one patient following Valsalva.

### Statistical analysis

Results are presented as mean ± standard deviation. Continuous variables were compared using the Wilcoxon signed-rank test or Mann–Whitney *U*-test for related or independent observations, respectively. Correlation between variables was expressed using the Spearman correlation coefficient (*r*_s_). A *P* < 0.05 was considered to be statistically significant for all tests.

## Results

All UEG recordings and respiratory recordings were analysable. Three blood pressure recordings were inadequate to allow analysis throughout the entire 60 s (2 during the resting period and 1 in the 60 s following termination of the Valsalva). A total of 22 UEG, 22 respiratory recordings, and 19 blood pressure recordings were analyzed. Throughout the protocol the pacing cycle length remained constant for all patients. The mean pacing cycle length used was 703 ± 80 ms.

### Activation recovery interval and blood pressure variability

**Figure 3:**
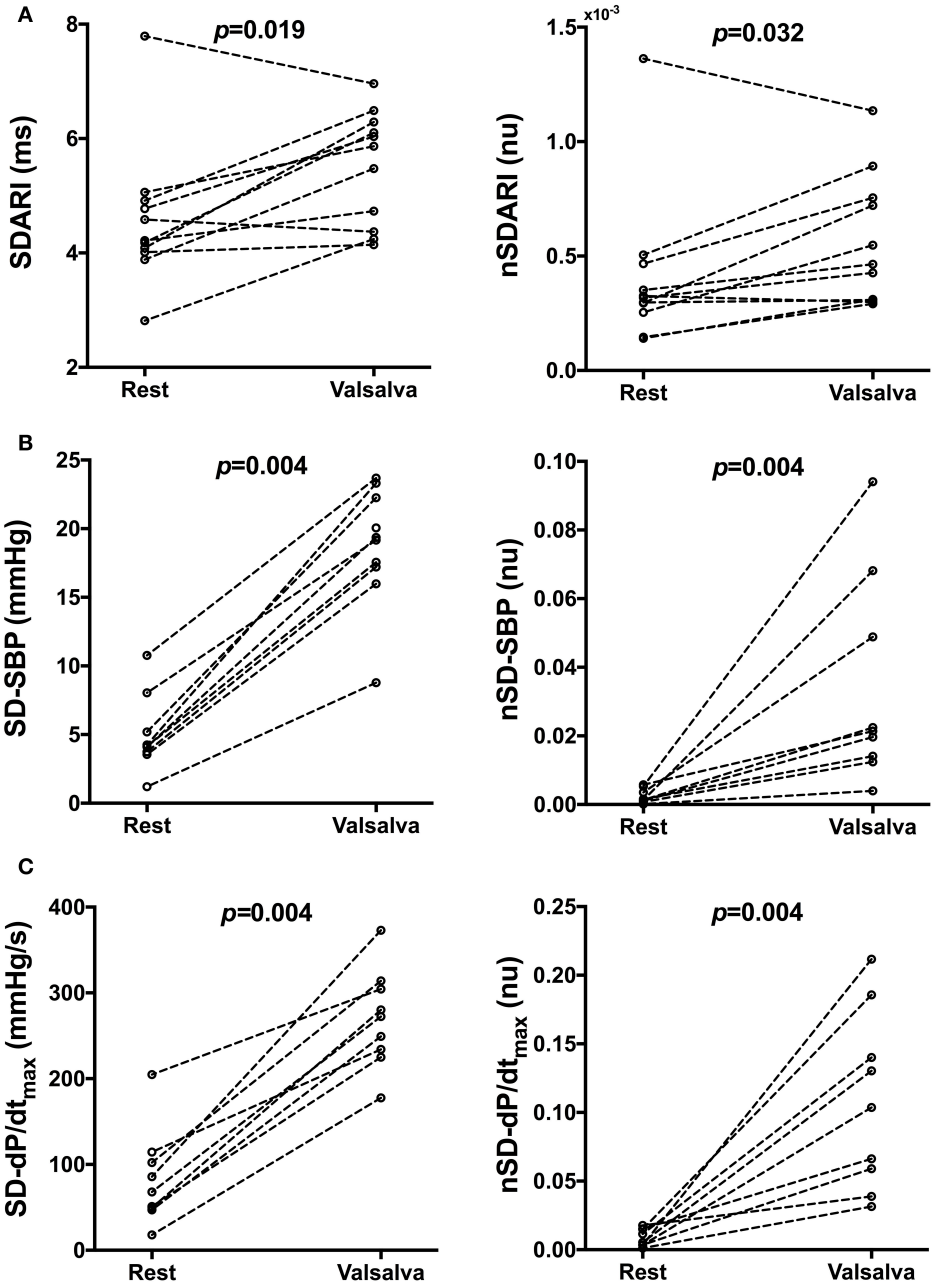
Individual beat-to-beat variability measures of **(A)** activation recovery intervals (ARIs), **(B)** systolic blood pressure (SBP) and **(C)** the maximum rate of systolic pressure increase (dP/dt_max_) at rest and following the Valsalva. SD, Standard deviation, nSD, Normalized SD.

Mean ARI, SBP, and dP/dt_max_ measurements during rest and following the Valsalva are reported in Table 2. Mean SDARI increased significantly following the Valsalva compared to the control period (*p* = 0.019). Mean nSDARI also increased significantly (*p* = 0.032). Figure 3A shows individual values of SDARI and nSDARI during control and following the Valsalva. As expected, mean ARI did not change between control and following stimulus (*p* = 0.147). There was no evidence of an increase in conduction variability following the Valsalva as assessed by AT-AT interval variability: AT-AT interval variability at rest (4.42 ± 0.41 ms) and following the Valsalva (4.52 ± 0.57 ms) was similar (*p* = 0.7).

**Table 2:** Mean activation recovery interval (ARI) and blood pressure measurements at rest and following the Valsalva.

	**Control**	**Post valsalva**	***P*-*value***
Mean ARI, ms	245.95 (±32.94)	244.75 (±33.63)	0.147
SDARI, ms	4.57 (±1.23)	5.52 (±0.99)	0.019
nSDARI, nu	4.06 × 10^4^ (±3.36 × 10^4^)	5.59 × 10^4^ (±2.82 × 10^4^)	0.032
Mean SBP, mmHg	113.42 (±19.13)	115.96 (±28.84)	0.426
SD-SBP, mmHg	4.99 (±2.80)	18.74 (±4.35)	0.004
nSD-SBP, nu	2.31 × 10^3^ (±2.05 × 10^3^)	3.74 × 10^2^ (±3.05 × 10^2^)	0.004
Mean dP/dt max, mmHg/s	959.11 (±471.54)	925.73 (±311.25)	0.652
SD-dP/dt max, mmHg/s	82.29 (±54.86)	276.96 (±58.17)	0.004
nSD-dP/dt max, nu	8.47 × 10^3^ (±6.34 × 10^3^)	11.3 (±6.34 × 10^2^)	0.004

*SD, Standard deviation; nSD, Normalized SD; SBP, Systolic blood pressure; dP/dt_max_, maximum rate of systolic pressure increase. Values are shown as means (±SD)*.

Mean SD-SBP increased following the Valsalva (*p* = 0.004). Mean nSD-SBP also significantly increased (*p* = 0.004). Figure 3B shows individual values of SD-SBP and nSD-SBP during control and following the Valsalva. Mean SD-dP/dt_max_ increased following the Valsalva (*p* = 0.004). Mean nSD-dP/dt_max_ also significantly increased (*p* = 0.004). Figure 3C shows individual values of SD-dP/dt_max_ and nSD-dP/dt_max_ during control and following the Valsalva. As per the behavior observed in mean ARI, there was no change in mean SBP (*p* = 0.426) nor mean dP/dt_max_ (*p* = 0.652).

Evaluation of the heart rate response for assessment of individuals autonomic response to the Valsalva was not possible due to the fixed paced cycle length. It has also been documented that heart failure patients may show an altered blood pressure response to the Valsalva without a fall in blood pressure during the strain phase known as a “square wave” blood pressure response (Felker et al., 2006). None of our patients exhibited this behavior (including the 2 patients with NYHA 3 class heart failure), all showing a substantial blood pressure drop in phase II of the Valsalva (Porter et al., 2017). To determine the significance of this blood pressure drop on the BBV of ARI in individual patients, a correlation analysis with the individual change from rest in SDARI and nSDARI was performed. Δ SDARI and Δ nSDARI showed no correlation with the degree of mechanical blood pressure drop (*p* = 0.958 and *p* = 0.689, respectively).

The behavior of SDARI in patients with ischaemic cardiomyopathy (ICM) (*n* = 6) vs. non-ischaemic cardiomyopathy (NICM) (*n* = 5) was similar. There was no observed difference in resting SDARI (4.15 ± 0.78 vs. 5.08 ± 1.56 ms, *p* = 0.537), nor SDARI post Valsalva (5.41 ± 0.76 vs. 5.65 ± 1.3 ms, *p* = 0.537) between patients with ICM vs. NICM.

### Frequency analysis

The respiratory frequency at rest (0.28 ± 0.08 Hz) and following termination of the Valsalva (0.26 ± 0.11 Hz) was similar (*p* = 0.473). This excluded the possibility of low frequency respiration producing associated low frequency changes in blood pressure and ARI at rest and post Valsalva.

**Figure 4:**
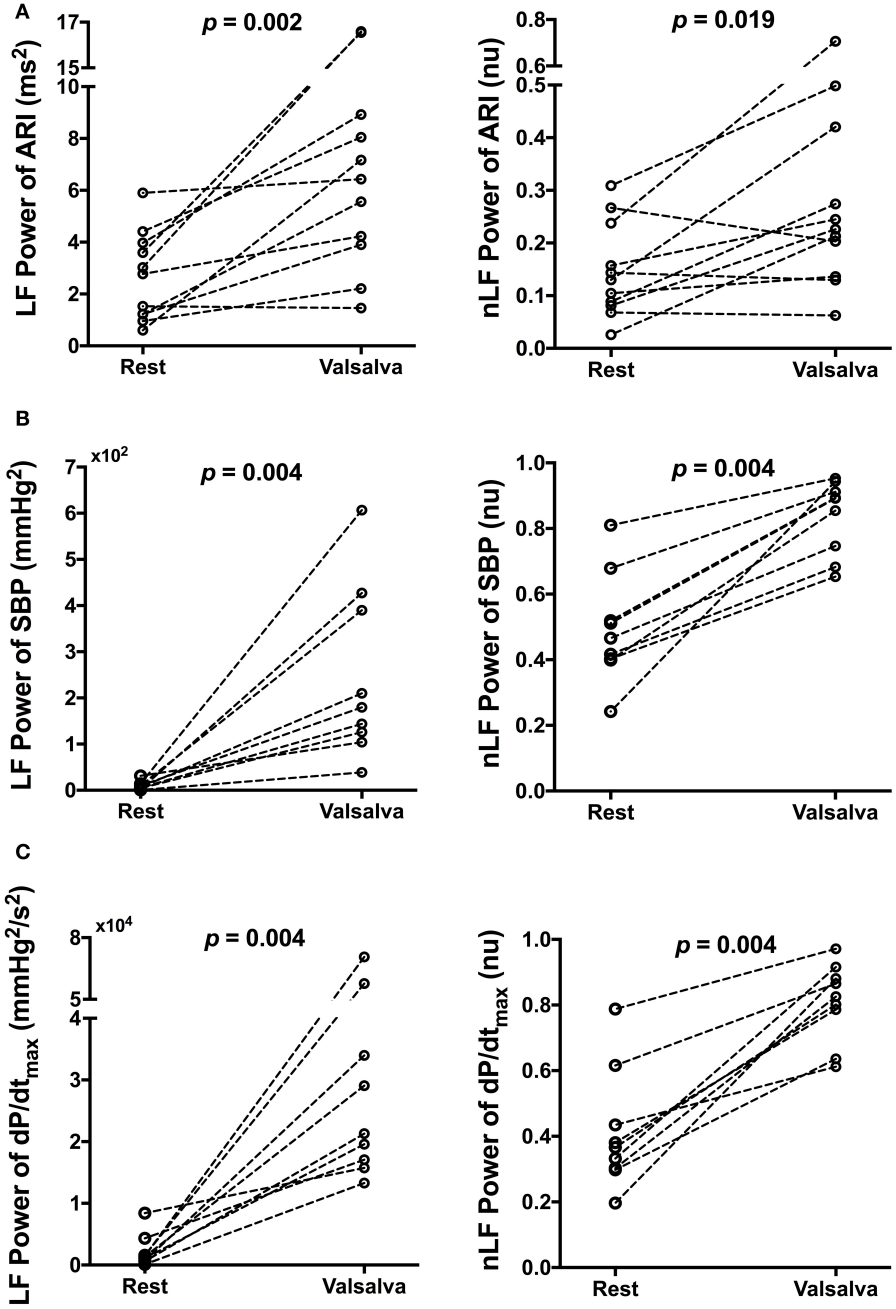
Effect of Valsalva on low frequency (LF) power and normalized LF (nLF) power of **(A)** activation recovery intervals (ARIs), **(B)** systolic blood pressure (SBP), and **(C)** the maximum rate of systolic pressure increase (dP/dt_max_) at rest and following the Valsalva.

Mean spectral energy measurements of ARI, SBP, and dP/dt_max_ during rest and following the Valsalva are reported in Table 3. Mean LF power of ARI (LF-ARI) significantly increased following the Valsalva (*p* = 0.002). When normalized the mean LF power of ARI continued to demonstrate a significant increase during the 60 s following the Valsalva when compared to control (*p* = 0.019). Figure 4A demonstrates the individual values of LF and nLF power of ARI during control and following the Valsalva. The Valsalva did not produce significant increases in the LF power of AT-AT intervals (rest: 0.73 ± 0.57 vs. post Valsalva: 2.76 ± 4.57, *p* = 0.365), nor in the normalized LF power of AT-AT intervals (rest: 0.04 ± 0.05 vs. post Valsalva: 0.13 ± 0.2, *p* = 0.365).

**Table 3:** Mean spectral analysis measurements of activation recovery interval (ARI), systolic blood pressure (SBP), and the maximum rate of systolic pressure increase (dP/dt_max_) at rest and following the Valsalva.

	**Control**	**Post valsalva**	***P*-value**
HF-ARI, ms^2^	10.61 (±6.52)	14.15 (±8.69)	0.007
nHF-ARI, nu	0.53 (±0.16)	0.51 (±0.2)	0.765
LF-ARI, ms^2^	2.65 (±1.7)	7.37 (±5.1)	0.002
nLF-ARI, nu	0.15 (±0.09)	0.28 (±0.19)	0.019
HF-SBP, mmHg^2^	7.88 (±7.04)	36.73 (±27.17)	0.004
nHF-SBP, nu	0.46 (±0.19)	0.17 (±0.12)	0.004
LF-SBP, mmHg^2^	9.45 (±9.15)	244.11 (±175.83)	0.004
nLF-SBP, nu	0.49 (±0.17)	0.84 (±0.11)	0.004
HF-dP/dt_max_, mmHg^2^/s^2^	2,794.07 (±3,354.78)	4,403.17 (±2,730.79)	0.129
nHF-dP/dt_max_, nu	0.46 (±0.2)	0.15 (±0.1)	0.004
LF-dP/dt_max_, mmHg^2^/s^2^	2,174.1 (±2,626.31)	31,417.85 (±19,097.79)	0.004
nLF-dP/dt_max_, nu	0.41 (±0.18)	0.82 (±0.12)	0.004

*HF, high frequency; nHF, normalized HF; LF, low frequency; nLF, normalized LF. Values are shown as means (±SD)*.

The Valsalva produced the same modulation of spectral energy measurements of blood pressure with that seen in ARI. Mean LF and nLF power of SBP and dP/dt_max_ significantly increased following the Valsalva (*p* = 0.004). Figures 4B,C demonstrate the individual values of LF and nLF power of SBP and dP/dt_max_ during control and following the Valsalva.

The behavior of LF-ARI in patients with ICM vs. NICM was similar. There was no observed difference in resting LF-ARI (1.98 ± 1.44 vs. 3.47 ± 1.77 ms^2^, *p* = 0.247), nor LF-ARI post Valsalva (6.98 ± 5.48 vs. 7.84 ± 5.18 ms^2^, *p* = 0.792) between patients with ICM vs. NICM.

**Figure 5:**
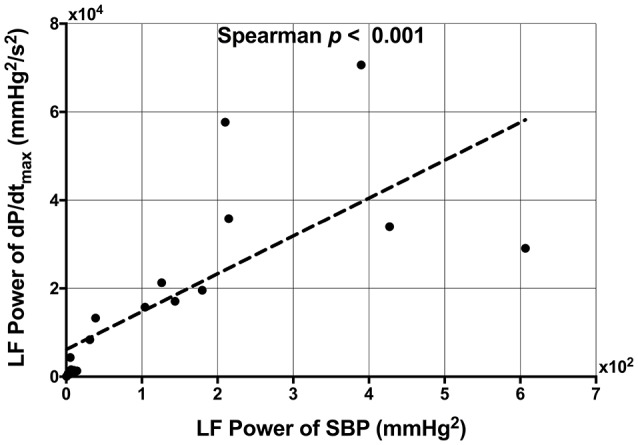
Scatterplot demonstrating the significant correlation between low frequency (LF) power of systolic blood pressure (SBP) and the LF power of the maximum rate of systolic pressure increase (dP/dt_max_).

**Figure 6:**
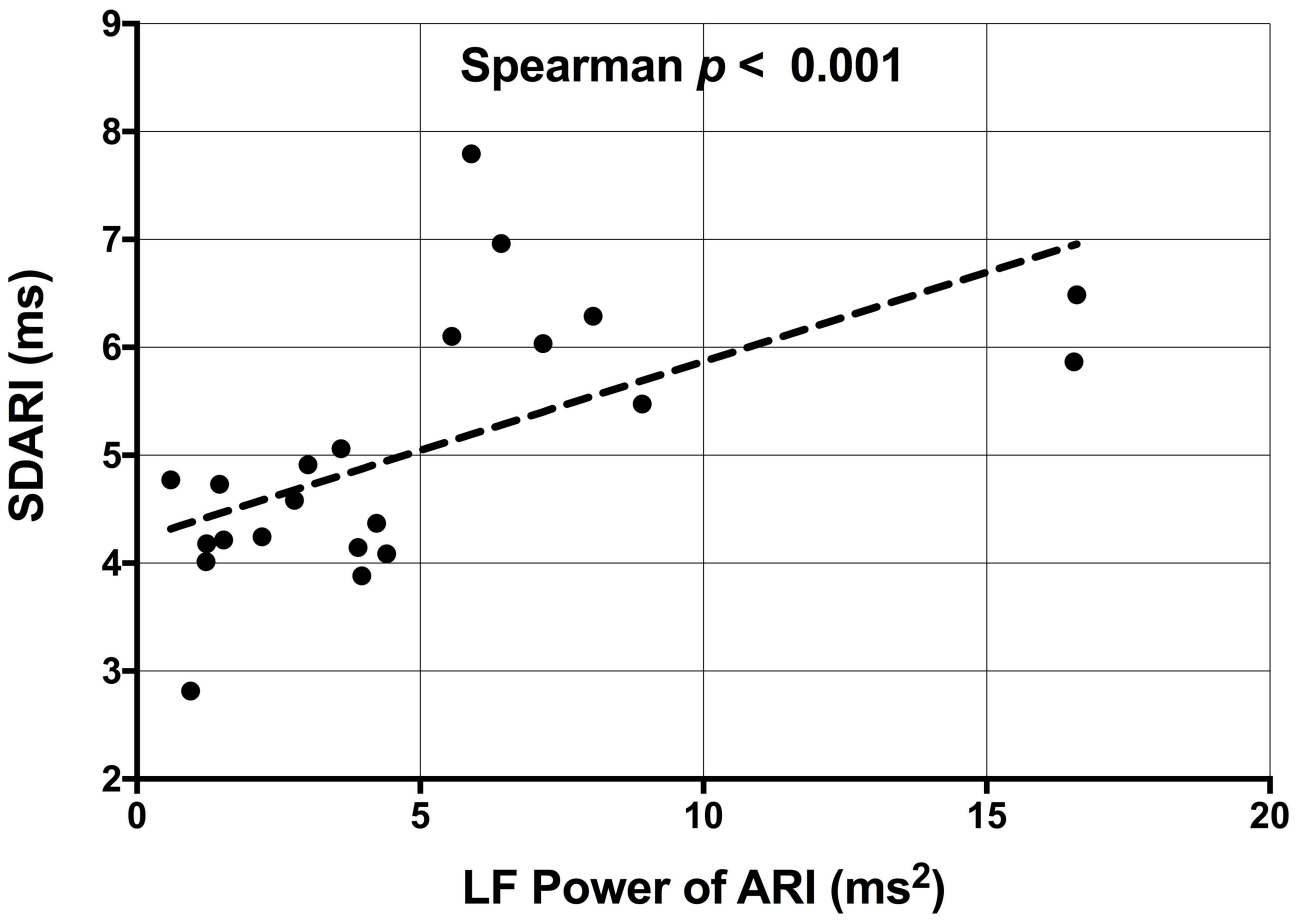
Scatterplot demonstrating the significant correlation between the low frequency (LF) power of activation recovery intervals (ARIs) and the beat-to-beat variability of ARI (SDARI).

A very strong positive correlation was found between LF-SBP and LF-dP/dt_max_ (*r*_s_ = 0.933, *n* = 19, *p* < 0.001) (Figure 5). A positive correlation was also found between LF-ARI and LF-SBP (*r*_s_ = 0.681, *n* = 19, *p* = 0.001) and between LF-ARI and LF-dP/dt_max_ (*r*_s_ = 0.623, *n* = 19, *p* = 0.004). There was a strong positive correlation between SDARI and LF-ARI (*r*_s_ = 0.679, *n* = 22, *p* < 0.001) (Figure 6). Importantly there was no apparent association between the hemodynamic response to the Valsalva (assessed by the blood pressure drop in phase II) and the increases in the individual oscillatory behavior of APD: Δ LF-ARI showed no correlation with the degree of mechanical blood pressure drop (*p* = 0.235).

## Discussion

We have investigated the oscillatory behavior of ventricular repolarization in the low frequency range (0.04–0.15 Hz) in response to a standard autonomic challenge (Valsalva maneuver). ARIs as a conventional surrogate for APD were recorded from the left ventricular epicardial lead of an implanted biventricular pacing device while pacing from the right ventricular lead. The main findings were: (1) BBV of APD (ARI) increased associated with an increase in BBV of SBP and dP/dt_max_ of systolic pressure, (2) the low frequency power of APD increased also associated with an increase in the low frequency power of SBP and dP/dt_max_, (3) the increase in beat-to-beat APD variability correlated with increasing low frequency power of APD.

Sympathetic nerve activity is organized in bursts in a range of frequencies including the so called low frequency (LF) range in the region of 0.04–0.15 Hz, i.e., approximately one every 10 s in humans (Pagani et al., 1986, 1997; Malliani et al., 1991; Furlan et al., 2000; Coote, 2001; Montano et al., 2009). Spectral analysis of blood pressure usually reveals a LF component. Fluctuations in blood pressure at this frequency (Mayer waves; Julien, 2006) are generally attributed to the response of peripheral vascular resistance to phasic sympathetic nerve input. BBV of heart rate also exhibits a LF component although the contribution of sympathetic activity remains a subject of discussion. Recently attention has been drawn to the presence of oscillations in the morphology of the T wave on the ECG in humans at approximately the 0.1 Hz frequency which are enhanced by sympathetic provocation (orthostatic challenge, exercise) and reduced by beta-adrenergic blockade (Rizas et al., 2014, 2016). These T wave oscillations have been attributed to oscillations in ventricular repolarization in response to LF sympathetic nerve input and assumed to reflect corresponding oscillations in ventricular APD. We have recently demonstrated the presence of LF oscillations of ventricular APD in humans (Hanson et al., 2014). In the present study we confirm the presence of these LF APD oscillations and demonstrate an increase following a conventional sympathetic provocation maneuver consistent with the findings of Rizas and colleagues on the ECG T wave (Rizas et al., 2014, 2016).

Extrapolation from our findings on BBV of APD with cycle length maintained constant to studies of QT variability is not straightforward. In physiological conditions QT variability is substantially influenced by heart rate variability due to the rapid and slow components of the cycle length dependence of APD (Franz et al., 1988; Zaza et al., 1991; Cabasson et al., 2012). QT variability when cycle length is held constant is considered to be due to both variation in APD and variation in activation pattern and hence conduction time (Baumert et al., 2016). In our studies, the Valsalva did not induce changes in the variability of conduction time nor in the low frequency oscillatory behavior of conduction.

BBV of repolarization has been demonstrated in a range of experimental models and humans (Zaniboni et al., 2000; Hinterseer et al., 2010; Tereshchenko et al., 2010). Exaggerated BBV of repolarization is known to be associated with arrhythmogenesis in animal models (Thomsen et al., 2004; Gallacher et al., 2007; Abi-Gerges et al., 2010; Jacobson et al., 2011) and in humans (Atiga et al., 1998; Haigney et al., 2004; Tereshchenko et al., 2009; Hinterseer et al., 2010; Sredniawa et al., 2012) and increase in the normal BBV in the timing of repolarization is associated with pro-arrhythmia (Baumert et al., 2016). Enhanced sympathetic activity has been shown to increase BBV of repolarization in canine ventricular myocytes (Johnson et al., 2013), the QT interval (Desai et al., 2004; Piccirillo et al., 2006) and recently of ventricular APD in humans (Porter et al., 2017). A previous study in normal subjects using spectral analysis of QT interval variability showed an increase in LF oscillation during both mental stress and exercise. This was attributed to a combined effect of increased sympathetic activity on RR interval variability and a direct effect on ventricular myocardium (Negoescu et al., 1993). A subsequent study using atrial pacing at a constant rate to eliminate RR interval variability confirmed that mental stress increased the LF power of QT variability. These results suggested a direct rate independent effect of enhanced sympathetic activity (Negoescu et al., 1997). The present study during constant steady state pacing extends these observations to the level of the ventricular action potential. We show that not only does a sympathetic challenge increase BBV of APD but also imparts a LF oscillation such that the BBV of APD waxes and wanes over a roughly 10 s cycle.

### Mechanisms

Oscillations of ventricular repolarization in the low frequency range independent of RR interval have been demonstrated in human endocardium (Hanson et al., 2014), body surface ECG recordings of the T-wave vector (Rizas et al., 2014, 2016, 2017) and in *in-silico* modeling studies (Pueyo et al., 2016). These rhythmic fluctuations are increased by enhanced sympathetic activity and reduced by beta-adrenergic blockade and occur in the spectral range of oscillations characteristic of sympathetic nerve activity (0.04–0.15 Hz). Modeling studies of the dynamics of the ventricular APD (Pueyo et al., 2016) suggest that the phasic nature of amplification with sympathetic stimulation is related to the different phosphorylation kinetics in response to beta-adrenergic stimulation of inward depolarizing calcium current (ICaL) and outward repolarizing potassium current (IKs) (Liu et al., 2012; Xie et al., 2013; Ruzsnavszky et al., 2014). The oscillatory behavior was markedly enhanced by incorporating interaction with beta adrenergic effects on myocardial stretch by calcium mechanisms and stretch activated channels, and also by incorporating calcium overload and downregulation of potassium channels, both of which are characteristic features of pathological hearts and remodeling and both known to promote the development of after-depolarizations and arrhythmias (Weiss et al., 2015).

Our study showing a correlation between the increase in low frequency patterning of APD and the increase in BBV of APD suggests a possible interaction between the underlying mechanisms. While a number of mechanism have been proposed as the cellular basis for BBV of repolarization, it is likely that spontaneous sarcoplasmic calcium release plays a fundamental role (Li et al., 2012; Johnson et al., 2013; Kim et al., 2013; Baumert et al., 2016). Calcium release from the sarcoplasmic reticulum varies on a beat-to-beat basis and in calcium overloaded cells may generate BBV of APD (Johnson et al., 2013). The apparently random nature of BBV favors a stochastic process, and stochastic variation in gating of a wide range of ion channels has been shown to influence BBV (Tanskanen et al., 2005; Heijman et al., 2013; Johnson et al., 2013). It is at present uncertain to what extent these effects observed in isolated cells may be operative in the whole heart due to electrotonic interaction between cells (Zaniboni et al., 2000; Baumert et al., 2016). However, in pathological hearts where cell coupling is reduced and in the presence of calcium overload and reduced repolarization reserve BBV may be arrhythmogenic by inducing early or late after-depolarizations or initiating re-entry. Modeling studies exploring the mechanisms of sympathetic enhancement of low frequency oscillation of APD showed an important contribution of accompanying mechanical changes mediated by stretch activated channels (Pueyo et al., 2016). Recent work in a canine model of chronic atrioventricular block and reduced repolarization reserve has shown that BBV in preload enhanced BBV of repolarization and was proarrhythmic. This effect was blocked by streptomycin therefore strongly suggesting a role of stretch activated channels in the modulation of BBV of APD (Stams et al., 2016).

### Clinical perspective

Risk stratification for the identification of patients at high risk of sudden cardiac death, particularly post MI, remains a major challenge. In view of the multiple mechanisms involved it is unlikely that a single test would prove sufficient and a combination of clinical characteristics with a selection of stratification tools may be more appropriate (Dagres and Hindricks, 2013). Oscillation of ventricular repolarization related to sympathetic activity, referred to as periodic repolarization dynamics (PRD) has been identified as a strong predictor of sudden cardiac death (Rizas et al., 2014, 2017) and is currently involved in a randomized prospective multicentre trial in 17 centers in Germany (Hamm et al., 2017). However, the mechanisms underlying PRD remain poorly understood. PRD provides incremental prognostic information to exercise induced T wave alternans (TWA) and is capable of detecting patients not identified by TWA (Rizas et al., 2014). The two may be complementary acting through different mechanism, PRD probably relates to low frequency sympathetic activity and TWA to high frequency oscillations related to calcium handling (Narayan et al., 2008). PRD may be obtained at rest whereas TWA requires exercise or invasive procedures to induce a heart rate increase to the region of 2 Hz. We have previously demonstrated the presence of oscillation of the ventricular APD at the sympathetic nerve frequency in humans (Hanson et al., 2014) and modeling studies identified a cellular mechanism related to the phosphorylation kinetics of ion channels (Pueyo et al., 2016). The present study extends our knowledge on mechanisms underlying the oscillatory behavior of repolarization at the level of the ventricular action potential oscillation in humans and a possible interaction with BBV of APD, an important proarrhythmic mechanism. Further studies investigating the oscillatory behavior of electrophysiological properties at the intact heart, tissue and cellular level may help refine its potential use in risk stratification as well as pointing toward novel therapeutic modalities.

### Limitations

An implanted biventricular pacing device was a basic requirement for the study which negated the possibility of incorporating a control group of subjects. Consequently, our findings are only directly applicable to patients with heart failure as we have not been able to study a population of patients with normal hearts. Similar studies of normal hearts would be of high importance to fully determine the significance of the observed changes in BBV of APD. Our study includes both patients with ischaemic and non-ischaemic cardiomyopathy. Although we observed changes in BBV of APD and also the low frequency behavior of APD in both groups, our study is underpowered to determine whether subtle differences in behavior exist between these populations. Although all of our patients had a typical haemodynamic response to the Valsalva, this may not be representative of the spectrum of NYHA classes of heart failure. Our study is underpowered to observe differences in behavior between various functional classes of heart failure. Our observations are confined to a single left ventricular epicardial site as it was only possible to record from one epicardial electrode whilst maintaining a constant cycle length through right ventricular pacing. In view of the regional variation in electrophysiological properties throughout the myocardium other regions may have yielded different results. Recordings were made during free breathing and respiration is known to affect repolarization lability. Due to a high prevalence of atrial fibrillation amongst our population of heart failure patients we chose RV pacing over atrial pacing to achieve fixed cycle length. Future studies would assess ARI variability during atrial pacing for further validation. Due to the fixed paced cycle length that is established throughout the protocol it is not possible to evaluate the heart rate response to the Valsalva and we are therefore not able to determine the Valsalva ratio which is a well-established measurement of the autonomic response.

## Conclusions

In patients with heart failure and implanted CRT-D devices physiological provocation to increase sympathetic activity induced oscillations of left ventricular APD in the low frequency range (0.04–0.15 Hz). Coherence analysis suggested an interaction between the low frequency oscillatory behavior of APD and the BBV. These observations provide insight at the level of the ventricular action potential into mechanisms underlying low frequency oscillation of repolarization derived from the ECG T-wave which have been shown to be exaggerated by sympathetic stimulation and are strongly predictive of sudden cardiac death in post MI patients. Further work aimed at unraveling mechanisms at the cellular level may help to elucidate the link to arrhythmogenesis and possibly point to therapeutic targets.

## Author contributions

BP, SvD, MB, JG, and PT conceived and designed the experiments. All authors took responsibility in collecting, analyzing, and interpreting the data. All authors contributed to drafting the manuscript.

### Conflict of interest statement

The authors declare that the research was conducted in the absence of any commercial or financial relationships that could be construed as a potential conflict of interest.
